# Anti-emetic Action of the Brain-Penetrating New Ghrelin Agonist, HM01, Alone and in Combination With the 5-HT_3_ Antagonist, Palonosetron and With the NK_1_ Antagonist, Netupitant, Against Cisplatin- and Motion-Induced Emesis in *Suncus murinus* (House Musk Shrew)

**DOI:** 10.3389/fphar.2018.00869

**Published:** 2018-08-06

**Authors:** John A. Rudd, Sze W. Chan, Man P. Ngan, Longlong Tu, Zengbing Lu, Claudio Giuliano, Emanuela Lovati, Claudio Pietra

**Affiliations:** ^1^Emesis Research Group, School of Biomedical Sciences, Faculty of Medicine, The Chinese University of Hong Kong, Shatin, Hong Kong; ^2^Brain and Mind Institute, The Chinese University of Hong Kong, Shatin, Hong Kong; ^3^School of Health Sciences, Caritas Institute of Higher Education, Tseung Kwan O New Town, Hong Kong; ^4^Helsinn Healthcare SA, Research and Development, Lugano, Switzerland

**Keywords:** ghrelin, HM01, nausea, emesis, chemotherapy, motion

## Abstract

Ghrelin has well-known activity to stimulate appetite and weight gain. Evidence suggests that ghrelin may also have effects in reducing chemotherapy-induced emesis via growth hormone secretagogue receptors (GHS-R1A) in the brain. However, it is not known whether the stimulation of GHS-R1A has broad inhibitory anti-emetic effects. In the present studies, we used *Suncus murinus* to investigate the potential of the new and novel orally bioavailable brain-penetrating GHS-R1A mimetic, HM01 (1-[(1S)-1-(2,3-dichloro-4-methoxyphenyl)ethyl]-3-methyl-3-[(4R)-1-Methyl-3,3-dimethyl-4-piperidyl]urea), to reduce emesis induced by a variety of emetic challenges. HM01 (1 to 30 mg/kg, p.o.) antagonized emesis induced by cisplatin (30 mg/kg, i.p.) and by motion (4 cm horizontal displacement, 1 Hz) but was ineffective against emesis induced by nicotine (5 mg/kg, s.c.) and copper sulfate (120 mg/kg by intragastric gavage). In other experiments, HM01 (3 mg/kg, p.o.) enhanced the anti-emetic control of a regimen of palonosetron (0.01 mg/kg, p.o.) alone and palonosetron (0.01 mg/kg p.o.) plus netupitant (1 mg/kg, p.o.). HM01 (10 mg/kg, p.o.) also had positive effects in increasing feeding and drinking in nicotine-treated animals, and it shortened the latency to drink in animals treated with cisplatin. These data indicate that brain-penetrating GHS-R1A agonists may have use alone and/or in combination with standard anti-emetic regimens for the treatment of chemotherapy-induced nausea and vomiting and motion sickness.

**Highlights:**

- The novel orally bioavailable brain-penetrating GHS-R1A agonist, HM01 (1-[(1S)-1-(2,3-dichloro-4-methoxyphenyl)ethyl]-3-methyl-3-[(4R)-1-Methyl-3,3-dimethyl-4-piperidyl]urea), antagonizes motion- and cisplatin-induced emesis.
- HM01 did not reduce emesis induced by nicotine or by intragastric copper sulfate.
- HM01 has positive effects on food consumption after treatment with nicotine.
- HM01 has synergistic effects against cisplatin when combined with palonosetron and palonosetron/netupitant regimens.
- It is suggested that GHS-R1A agonists may be protective against chemotherapy-induced nausea and vomiting in combination with traditional anti-emetics and against motion-induced emesis.

## Introduction

Cancer treatment with agents such as cisplatin has a well-known association with nausea and emesis via mechanisms that are believed to predominantly involve the release of 5-hydroytryptamine (5-HT) in the gastrointestinal tract, which activates vagal afferents, but also a release of substance P in the brainstem to activate tachykinin NK_1_ receptors. Inflammatory mediators may also be involved, and 5-HT_3_ receptors may play a role in the dorsal vagal complex of the brainstem (Rudd and Andrews, 2004; Hesketh, 2008; Andrews and Rudd, 2015). Guidelines for nausea and emesis control involve a combination of 5-HT_3_ and tachykinin NK_1_ receptor antagonists plus a glucocorticoid such as dexamethasone (Roila et al., 2017). Second-generation 5-HT_3_ receptor antagonists (e.g., palonosetron) and NK_1_ receptor antagonists (e.g., netupitant) are now available and have greater potency and/or more favorable pharmacokinetics than older-generation compounds, which is reflected in their superior clinical efficacy (Hesketh et al., 2014; Navari, 2015; Rudd et al., 2016). Yet despite these advances, a proportion of patients still have inadequate protection from chemotherapy-induced nausea and emesis (Gralla et al., 2014; Van den Brande et al., 2014).

Ghrelin is a peptide that stimulates feeding and gastrointestinal motility via growth hormone secretagogue receptors (GHS-R1A) (Ogawa et al., 2012; Sanger et al., 2013; Muller et al., 2015). We previously demonstrated that ghrelin could antagonize cisplatin-induced acute emesis in ferrets via central actions in the brain (Rudd et al., 2006). Since then, studies in man have shown that ghrelin can improve the control of chemotherapy-induced nausea and emesis and increase appetite when combined with the 5-HT_3_ receptor antagonist, ramosetron (Hiura et al., 2012). Ghrelin mimetics may be useful in other situations to treat nausea and emesis. Anamorelin has shown particular benefit in patients with cancer cachexia (Currow and Abernethy, 2014); relamorelin has shown benefit in patients with diabetic gastroparesis via enhancement of gastric emptying and reduction of emesis (Camilleri and Acosta, 2015); and ulimorelin has shown benefit in patients with postoperative ileus, in whom it reduced nausea and emesis (Shaw et al., 2013).

**FIGURE 1:**
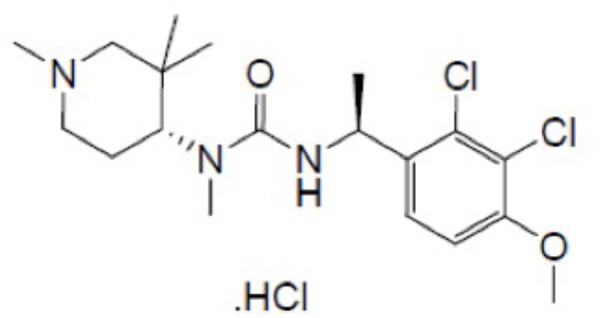
The chemical structure of HM01.

Surprisingly, no studies have been conducted to determine whether ghrelin or ghrelin mimetics have broad inhibitory anti-emetic properties. In this study, therefore, we investigated the potential of the new and recently synthesized orally bioavailable brain-penetrating ghrelin mimetic – HM01 (1-[(1S)-1-(2,3-dichloro-4-methoxyphenyl)ethyl]-3-methyl-3-[(4R)-1-Methyl-3,3-dimethyl-4-piperidyl]urea; **Figure 1**) – to inhibit emesis induced by nicotine, which is presumed to induce emesis via central mechanisms that involve the area postrema and/or autonomic nervous system (Laffan and Borison, 1957; Beleslin and Krstic, 1987), and to inhibit emesis induced by intragastric copper sulfate, which is presumed to induce emesis via peripheral vagal and splanchnic pathways from the gastrointestinal tract (Wang and Borison, 1952; Kan et al., 2006). We also investigated the potential of HM01 to antagonize emesis induced by motion, which involves sensory conflict and mechanisms that traverse the brainstem via the vestibular nuclei (Oman, 2012; Bertolini and Straumann, 2016).

It is feasible that ghrelin mimetics could be used in combination with standard anti-emetics for the treatment of chemotherapy-induced nausea and emesis, so we evaluated HM01 for the potential to antagonize cisplatin-induced emesis, both alone and in combination with palonosetron and/or netupitant (Hesketh et al., 2014). Ghrelin and HM01 are well known to increase appetite and food consumption (Karasawa et al., 2014; Muller et al., 2015; Villars et al., 2017). Therefore, most experiments in our study simultaneously assessed drug action on food and water consumption. We envisaged that an ability of a treatment to increase feeding during or after emetic treatments might provide an index to indicate the potential to reduce nausea. Common laboratory animals (e.g., mice, rats) are incapable of vomiting, so these studies used *Suncus murinus*, a species commonly used in research into mechanisms of acute chemotherapy- and motion-induced emesis (Sam et al., 2003; Percie du Sert et al., 2010). The amino acid sequence and cDNA sequence of ghrelin and its mRNA distribution in various tissues has been reported in this species (Ishida et al., 2009; Suzuki et al., 2012). Moreover, the role of ghrelin in the mechanisms of gastrointestinal motility and gastric acid production has also been characterized in *Suncus murinus* (Miyano et al., 2013; Mondal et al., 2013). It was thought that these studies would provide more insight into the role of GHS-R1A in the mechanism of emesis control necessary for the development of ghrelin mimetics as novel anti-emetic drugs.

## Materials and Methods

### Animals

Male *Suncus murinus* (45–65 g) were obtained from the Chinese University of Hong Kong and housed in a temperature-controlled room at 24°C ± 1°C under artificial lighting, with the lights on between 0600 and 1800 h. Humidity was maintained at 50% ± 5%. Water and dry cat chow pellets (Feline Diet 5003, PMI Feeds, St. Louis, MO, United States) were given *ad libitum*. All experiments were conducted under license from the Government of Hong Kong SAR and with permission from the Animal Experimentation Ethics Committee, The Chinese University of Hong Kong.

### Assessment of the Anti-emetic Potential of HM01

One day before experimentation, the animals were transferred to an observation room with controlled lighting (15 ± 2 Lux) and habituated to clear Perplex observation chambers (21 cm × 14 cm × 13 cm). Food and water were withdrawn immediately before the administration of HM01 (1 to 30 mg/kg, p.o.) or vehicle (distilled water, 2 ml/kg, p.o.). Sixty minutes later, the animals were administered nicotine (5 mg/kg, s.c.), copper sulfate pentahydrate (120 mg/kg, intragastric) or cisplatin (30 mg/kg, i.p.). Food and water were re-introduced 4 h after administration of the test emetogen. The animals’ behavior was recorded by an overhead camera (Panasonic WV-CP460/P; Panasonic, Yokahoma, Japan), and data were stored on a digital video recorder (Everfocus EDSR900; Everfocus, Taipei, Taiwan); food and water consumption were recorded during the 4- to 24-h period. In experiments involving provocative motion (1 Hz, 4 cm horizontal displacement for 10 min), the animals were observed for 10 min without assessment of food and water consumption.

### Assessment of the Anti-emetic Potential of HM01 in Combination With Palonosetron and/or Netupitant Against Cisplatin-Induced Emesis

Experiments were conducted to determine the potential of palonosetron (0.01–1 mg/k, p.o.) and netupitant (0.1–1 mg/kg, p.o.) to antagonize emesis induced by cisplatin (30 mg/kg, i.p.). The protocol was exactly as stated above for the drug-induced emesis studies. The data were inspected to determine the doses of the antagonists to reduce emesis by approximately 50–60%; similarly, the dose of HM01 that reduced cisplatin-induced emesis was calculated from the drug-induced emesis experiments. Thereafter, palonosetron (0.01 mg/kg, p.o.) and/or netupitant (1 mg/kg, p.o.) were combined with HM01 (3 mg/kg, p.o.) and administered 1 h before cisplatin (30 mg/kg, i.p.); the control animals were treated with vehicle (distilled water, 2 ml/kg, p.o.). Behavior and food and water consumption were measured as stated above.

### Drug Formulation

HM01 (1-[(1S)-1-(2,3-dichloro-4-methoxyphenyl)ethyl]-3-methyl-3-[(4R)-1-Methyl-3,3-dimethyl-4-piperidyl]urea; **Figure 1**) was obtained from Sundia, China. Palonosetron hydrochloride and netupitant were obtained from Helsinn Advanced Synthesis SA, Switzerland. Cisplatin was obtained from Sigma-Aldrich, St. Louis, MO, United States. (-)-Nicotine di-D-tartrate was obtained from Research Biochemicals, Inc., United States. Copper sulfate pentahydrate was obtained from British Drug Houses, United Kingdom. Cisplatin was dissolved in 0.9% w/v saline solution by gentle warming and adjusted to pH 4 with 0.1 N HCl. All other drugs were dissolved in distilled water. Doses are expressed as the free base.

### Measurement of Emesis

Emesis was characterized by rhythmical abdominal contractions that were either associated with the forceful oral expulsion of solid or liquid material from the gastrointestinal tract (i.e., vomiting) or not associated with the passage of material (i.e., retching movements). Consecutive episodes of retching and/or vomiting were considered separate when the animal changed its location in the observation cage, or when the interval between episodes exceeded 2 s (Rudd et al., 1999). At the end of the observation periods, the animals were killed with an intraperitoneal injection of pentobarbitone sodium (80 mg/kg).

### Experimental Design and Data Analysis

The treatments were randomized and administered following a Latin square design. The investigators were blinded to the treatments. GraphPad Prism 5.0a (GraphPad Software, La Jolla, CA, United States) was used to perform curve fitting (to determine ID_50_ values) and statistical comparisons. Retching, vomiting, body weight and food and water consumption data were analyzed using a one-way analysis of variance followed by Bonferroni’s or Dunnett’s multiple comparison tests. Latency data were analyzed using a Kruskal–Wallis test followed by Dunn’s multiple comparison tests. When an animal failed to retch or vomit, or to eat or drink, a latency value equal to the test period observation time was used to perform the statistical analysis. The results are expressed as the mean ± SEM unless otherwise stated. ID_50_ values were calculated from responses expressed as a percentage of the control data. In all cases, differences between treatment groups were considered significant when the *P*-value was less than 0.05.

## Results

### Anti-emetic Potential of HM01 Against Emesis Induced by Nicotine, Copper Sulfate, Cisplatin and Motion and Associated Changes in Food and Water Consumption and Body Weight

#### Nicotine

**FIGURE 2:**
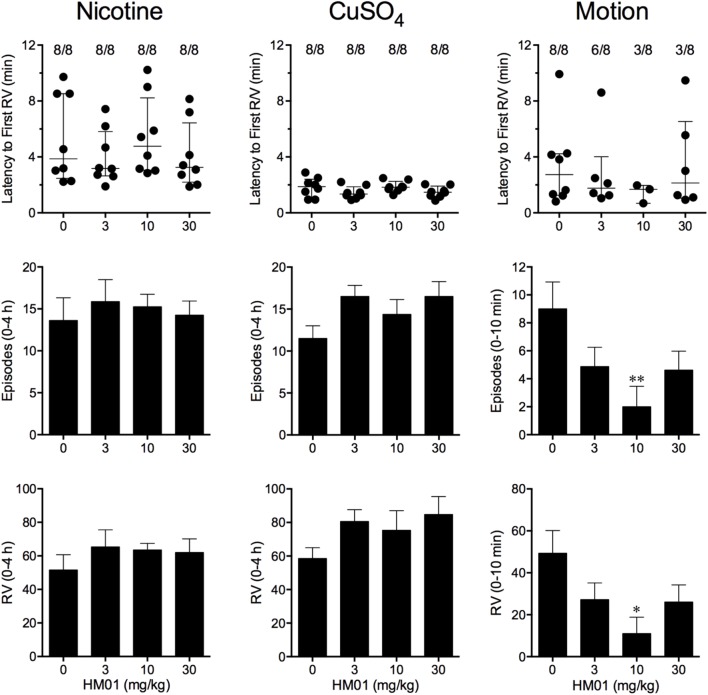
Effect of HM01 (3–30 mg/kg, p.o.) administered as a 60 min pretreatment on nicotine (0.5 mg/kg, s.c.)-, copper sulfate (120 mg/kg, i.g.)- and motion (1Hz, 4 cm displacement, 10 min)-induced emesis in *Suncus murinus*. Individual animal latencies to the first episode of retching and/or vomiting, and lines indicating medians with interquartile ranges are shown, as are the number of animals exhibiting retching and/or vomiting out of the number of animals tested. The total number of retches + vomits occurring during the respective observation periods are shown as the mean ± SEM. Significant differences relative to the vehicle-treated animals are indicated as ^∗^*P* < 0.05, ^∗∗^*P* < 0.01 (Kruskal–Wallis test or one-way ANOVA followed by appropriate *post hoc* testing, as appropriate).

**FIGURE 3:**
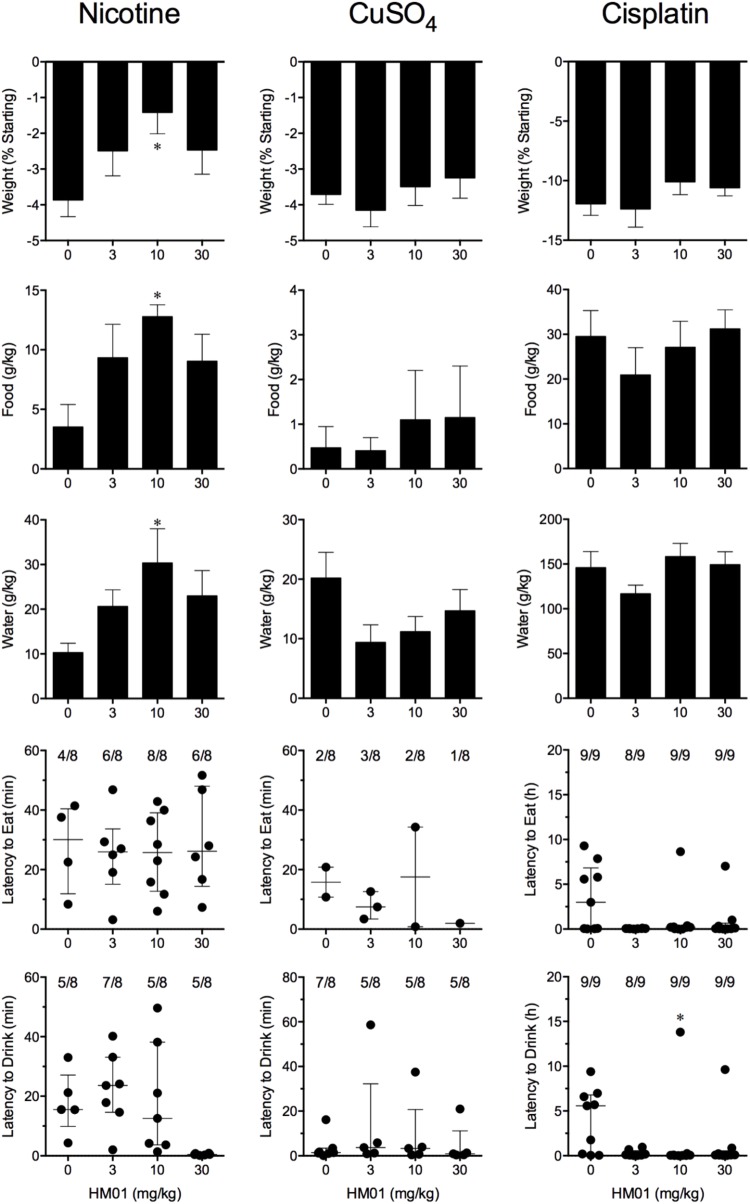
Effect of HM01 (3–30 mg/kg, p.o.) administered as a 60 min pretreatment on nicotine (0.5 mg/kg, s.c.)-, copper sulfate (120 mg/kg, i.g.)- and cisplatin (30 mg/kg, i.p.)-induced changes of food and water consumption in *Suncus murinus*. Individual animal latencies to the first episode of eating or drinking, and lines indicating medians with interquartile ranges are shown, as are the number of animals exhibiting eating or drinking out of the number of animals tested. Results represents the mean ± SEM. Significant differences relative to the vehicle-treated animals are indicated as ^∗^*P* < 0.05 (Kruskal–Wallis test or one-way ANOVA followed by appropriate *post hoc* testing, as appropriate).

Nicotine induced emesis in animals treated with vehicle (0 mg/kg) after a median latency of 3.9 min (quartiles, 2.5 and 8.5 min; **Figure 2**). The emetic response comprised 48.6 ± 8.4 retches and 3.0 ± 0.8 vomits in 13.6 ± 2.7 episodes. HM01 did not modify any of the retching and/or vomiting parameters compared with controls (**Figure 2**). At the end of the experiments, the animals treated with nicotine and vehicle had lost 3.9% ± 0.5% of their starting body weight (**Figure 3**). HM01 at 10 mg/kg caused a significant reduction in nicotine-induced weight loss (approximately 64%; *P* < 0.05; **Figure 3**). The animals treated with nicotine vehicle consumed 3.5 ± 1.9 and 10.3 ± 2.1 g/kg of food and water, respectively, during the 4- to 24-h period (**Figure 3**). HM01 caused a significant increase (*P* < 0.05) in food and water consumption. The effects were maximal at 10 mg/kg, at which a 261.7% increase in food consumption and a 194.0% increase in water consumption were recorded (*P* < 0.05; **Figure 3**). However, HM01 did not affect the latency of the retching or vomiting or the latency of eating or drinking (**Figures 2**, **3**).

#### Copper Sulfate

Copper sulfate induced emesis in animals treated with vehicle (0 mg/kg) after a median latency of 1.9 min (quartiles, 1.1 and 2.4 min; **Figure 2**). The emetic response comprised 52.0 ± 5.9 retches and 6.5 ± 0.8 vomits in 11.5 ± 1.5 episodes. HM01 did not act to modify any of the retching and/or vomiting parameters compared with controls (*P* > 0.05; **Figure 2**). The control animals treated with copper sulfate and vehicle lost 3.7% ± 0.3% of their starting body weight (**Figure 3**). HM01 did not modify the copper sulfate–induced weight loss (*P* > 0.05; **Figure 3**). The copper sulfate control animals consumed 0.5 ± 0.5 and 20.2 ± 4.3 g/kg of food and water, respectively; only two of the eight animals ate, and seven of the eight drank. HM01 did not significantly modify food or water consumption (**Figure 3**). HM01 also failed to affect the latencies of the retching or vomiting or the latency of eating or drinking (**Figures 2**, **3**).

#### Motion

Motion induced emesis in animals treated with vehicle (0 mg/kg) after a median latency of 2.7 min (quartiles, 1.2 and 4.2 min; **Figure 2**). The emetic response comprised 49.4 ± 10.8 retches + vomits during the 10-min observation period (**Figure 2**). HM01 appeared to have a ‘U’-shaped dose-response curve; the 10-mg/kg dose significantly reduced the retching and vomiting response by 77.7% (*P* < 0.05), but it did not affect the latency to the first retch or vomit (*P* > 0.05; **Figure 2**). Food and water consumption parameters were not recorded during this experiment.

#### Cisplatin

**FIGURE 4:**
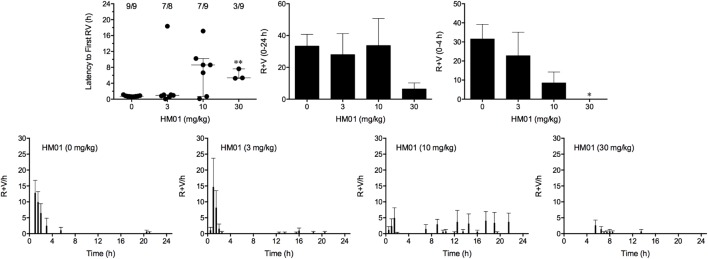
Effect of HM01 (3–30 mg/kg, p.o.) administered as a 60 min pretreatment on cisplatin (30 mg/kg, i.p.)-induced emesis in *Suncus murinus*. Individual animal latencies to the first episode of retching and/or vomiting, and lines indicating medians with interquartile ranges are shown, as are the number of animals exhibiting retching and/or vomiting out of the number of animals tested. The total number of retches + vomits occurring during the respective observation periods are shown as the mean ± SEM. Significant differences relative to the vehicle-treated animals are indicated as ^∗^*P* < 0.05, ^∗∗^*P* < 0.01 (Kruskal–Wallis test or one-way ANOVA followed by appropriate *post hoc* testing, as appropriate).

Cisplatin induced emesis in animals treated with vehicle (0 mg/kg) after a median latency of 0.7 h (quartiles, 0.7 and 0.9 h; **Figure 4**). The emetic response comprised 31.6 ± 7.7 retches + vomits during the first 4-h period; 33.4 ± 7.3 retches + vomits occurred during the entire 24-h observation period (**Figure 4**). HM01 reduced the retching + vomiting response during the first 4-h period in a dose-dependent manner, with an ID_50_ value of 6.8 ± 3.4 mg/kg; the highest dose of 30 mg/kg completely prevented emesis during this period in all nine animals (*P* < 0.05). The highest dose of HM01 also reduced retching and vomiting during the entire 24-h period by 80.1%; this reduction was not statistically significant, but six of the nine animals were protected completely (**Figure 4**); the latency to the first retching and/or vomiting episode was significantly delayed by approximately 4.6 h (*P* < 0.05; **Figure 4**). The control animals treated with cisplatin and vehicle lost 12.0% ± 1.0% of their starting body weight, but this was not affected by treatment with HM01 (**Figure 3**). During the observation period, the controls also ate 29.5 ± 5.8 g of food and consumed 146.1 ± 17.8 ml/kg of water (**Figure 3**). The latency to eat after food was introduced into the cage was 3.0 h (quartiles, 0.0 and 9.3 h); HM01 appeared to reduce the latency, but the effect was not statistically significant (**Figure 3**). The latency to drink after water was reintroduced into the cage was 5.6 h (quartiles, 0.1 and 6.8 h), and HM01 reduced the latency significantly at 10 mg/kg (*P* < 0.05; **Figure 3**).

### Anti-emetic Potential of HM01 in Combination With Oral Palonosetron and/or Oral Netupitant Against Cisplatin-Induced Emesis and Associated Changes in Food and Water Consumption and Body Weight

To conduct these experiments, it was necessary to first determine the anti-emetic potential of palonosetron and netupitant alone before designing the combination experiment with HM01.

### Effect of Palonosetron Alone Against Cisplatin

**FIGURE 5:**
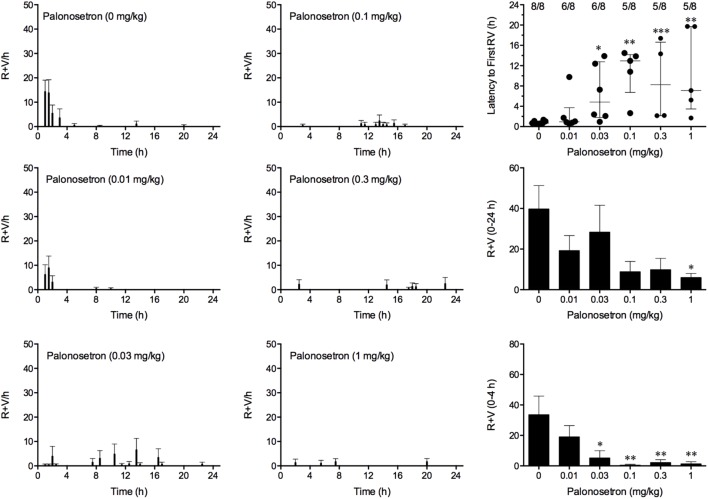
Effect of palonosetron (0.01–1 mg/kg, p.o.) administered as a 60 min pretreatment on cisplatin (30 mg/kg, i.p.)-induced emesis in *Suncus murinus*. Individual animal latencies to the first episode of retching and/or vomiting, and lines indicating medians with interquartile ranges are shown, as are the number of animals exhibiting retching and/or vomiting out of the number of animals tested. The total number of retches + vomits occurring during the respective observation periods are shown as the mean ± SEM. Significant differences relative to the vehicle-treated animals are indicated as ^∗^*P* < 0.05, ^∗∗^*P* < 0.01, ^∗∗∗^*P* < 0.001 (Kruskal–Wallis test or one-way ANOVA followed by appropriate *post hoc* testing, as appropriate).

**FIGURE 6:**
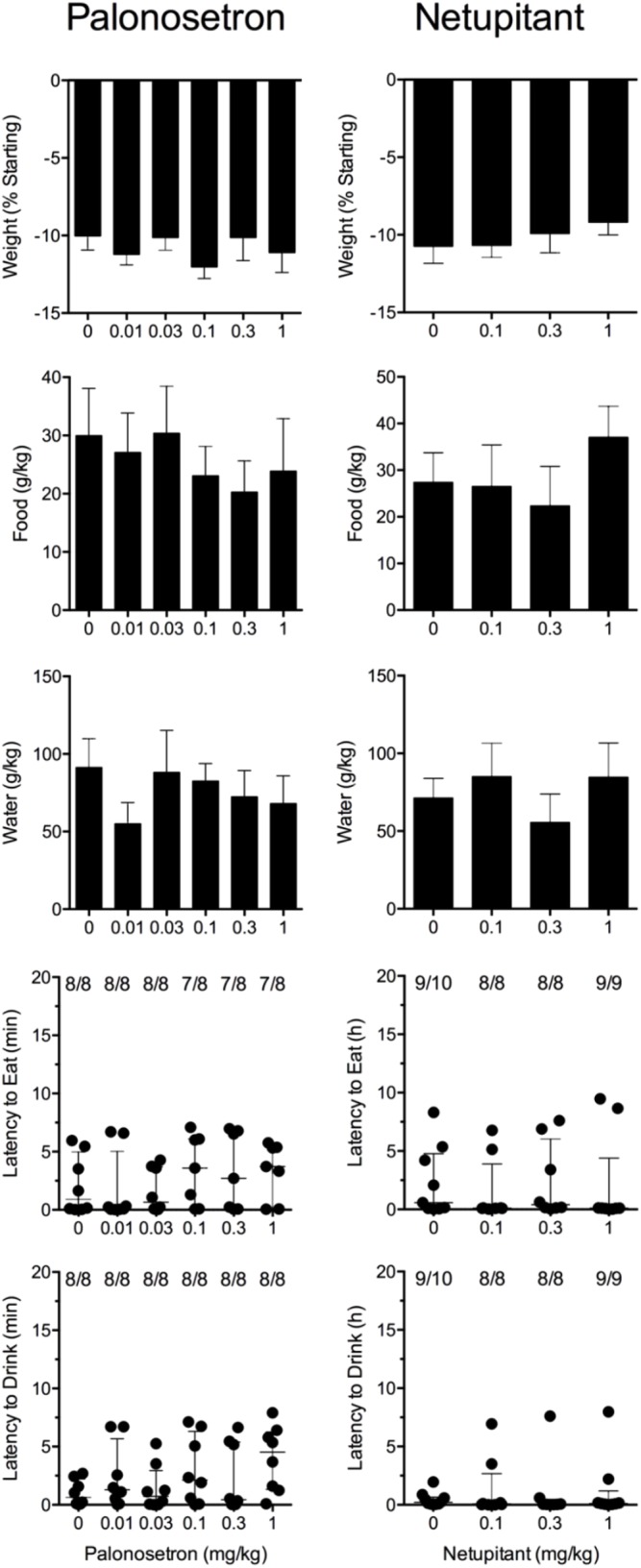
Effect of palonosetron (0.01–1 mg/kg, p.o.) and netupitant (0.1–1 mg/kg, p.o.) administered as a 60 min pretreatment on cisplatin (30 mg/kg, i.p.)-induced changes of food and water consumption in *Suncus murinus*. Individual animal latencies to the first episode of eating or drinking, and lines indicating medians with interquartile ranges are shown, as are the number of animals exhibiting eating or drinking out of the number of animals tested. Results represents the mean ± SEM. There were no significant differences relative to vehicle-treated animals (Kruskal–Wallis test or one-way ANOVA).

Cisplatin induced emesis in animals treated with vehicle (0 mg/kg) after a median latency of 0.7 h (quartiles, 0.6 and 1.1 h; **Figure 5**). The emetic response comprised 33.6 ± 12.2 retches + vomits during the first 4-h period; 39.8.4 ± 11.5 retches + vomits occurred during the entire 24-h observation period (**Figure 5**). Palonosetron reduced the retching + vomiting response during the first 4-h period in a dose-dependent manner, with an ID_50_ value of 9.8 ± 3.9 μg/kg; the highest dose of 1 mg/kg completely prevented emesis in seven of the eight animals (*P* < 0.05). The highest dose of palonosetron also reduced retching and vomiting during the entire 24-h period by 84.9% (*P* < 0.05); three of the eight animals were protected completely (**Figure 5**). Palonosetron also produced a dose-related effect that delayed the onset of the first retching and/or vomiting episode: at 0.03 mg/kg, there was a delay of 4.1 h (*P* < 0.5), and at 1 mg/kg, the delay increased to 6.4 h (*P* < 0.01). The control animals treated with cisplatin and vehicle lost 10.0% ± 0.9% of their starting body weight, but this was not affected by treatment with palonosetron (**Figure 6**). During the observation period, the controls also ate 30.0 ± 8.1 g/kg of food and consumed 91.3 ± 18.5 ml/kg of water (**Figure 6**). The latency to eat after food was reintroduced into the cage was 0.9 h (quartiles, 0.1 and 6.0 h). The latency to drink after water was reintroduced into the cage was 0.6 h (quartiles, 0.1 and 2.7 h). Palonosetron did not affect the amount of food or water consumed or the latencies to eat or drink (**Figure 6**).

### Effect of Netupitant Alone Against Cisplatin

**FIGURE 7:**
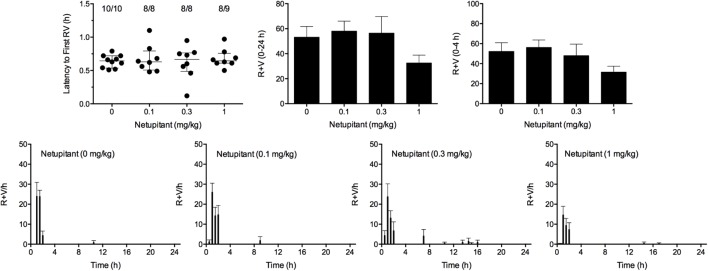
Effect of netupitant (0.1–1 mg/kg, p.o.) administered as a 60 min pretreatment on cisplatin (30 mg/kg, i.p.)-induced emesis in *Suncus murinus*. Individual animal latencies to the first episode of retching and/or vomiting, and lines indicating medians with interquartile ranges are shown, as are the number of animals exhibiting retching and/or vomiting out of the number of animals tested. The total number of retches + vomits occurring during the respective observation periods are shown as the mean ± SEM. There were no significant differences relative to vehicle-treated animals (Kruskal–Wallis test or one-way ANOVA).

Cisplatin induced emesis in animals treated with vehicle (0 mg/kg) after a median latency of 0.7 h (quartiles, 0.5 and 0.8 h; **Figure 7**). The emetic response comprised 52.3 ± 8.7 retches + vomits during the first 4-h period; 53.2 ± 8.5 retches + vomits occurred during the entire 24-h observation period (**Figure 7**). Netupitant resulted in a trend to decrease the retching and vomiting response during the first 4-h period and the entire 24-h period; the highest dose of 1 mg/kg reduced the respective responses by 39.5 and 38.8%, but none of the reductions were statistically significant. It was not possible to calculate an ID_50_ value from the data. The control animals treated with cisplatin and vehicle lost 10.7% ± 1.1% of their starting body weight, but this was not affected by treatment with netupitant (**Figure 6**). During the observation period, the controls also ate 27.4 ± 6.3 g/kg of food and consumed 71.3 ± 12.6 ml/kg of water (**Figure 6**). Netupitant did not affect the amount of food or water consumed, the latencies to the first retch or vomit, or the latencies to eat or drink (**Figure 6**).

### Effect of the Combination of Palonosetron, Netupitant and HM01 Against Cisplatin

**FIGURE 8:**
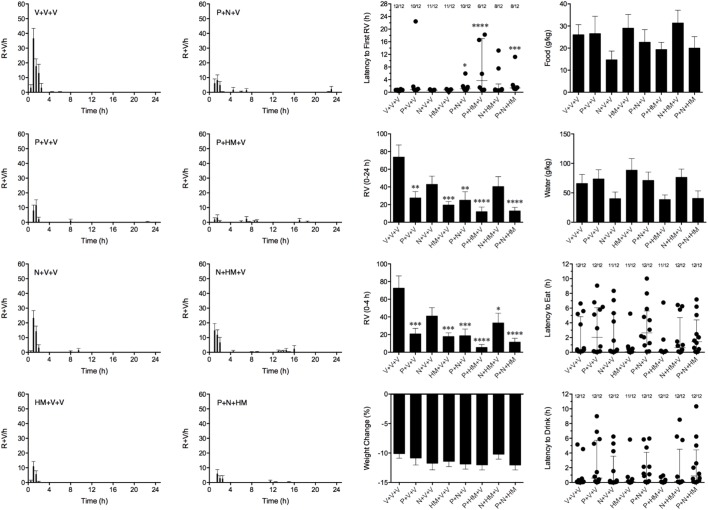
Effect of HM01 (HM) in combination of palonosetron (P) and/or netupitant (N) on cisplatin (30 mg/kg, i.p.)-induced emesis and associated changes in food and water consumption and body weight in *Suncus murinus*. Individual animal latencies to the first episode of eating, drinking, retching and/or vomiting, and lines indicating medians with interquartile ranges are shown, as are the number of animals exhibiting eating, drinking, retching and/or vomiting out of the number of animals tested. Results represents the mean ± SEM. Significant differences relative to the vehicle (V)-treated animals are indicated as ^∗^*P* < 0.05, ^∗∗^*P* < 0.01, ^∗∗∗^*P* < 0.001, ^∗∗∗∗^*P* < 0.0001 (Kruskal–Wallis test or one-way ANOVA followed by appropriate *post hoc* testing, as appropriate).

We aimed to combine palonosetron, netupitant and HM01 at oral doses that had been shown to reduce emesis by approximately 40%. Therefore, in these studies, palonosetron was used at 0.01 mg/kg, netupitant was used at 1 mg/kg and HM01 was used at 3 mg/kg. In these studies, cisplatin induced emesis in animals treated with vehicle (V+V+V; 0 mg/kg) after a median latency of 0.6 h (quartiles, 0.4 and 0.8 h; **Figure 7**). The emetic response comprised 72.6 ± 13.8 retches + vomits during the first 4-h period; 73.8 ± 13.7 retches + vomits occurred during the entire 24-h observation period (**Figure 8**). As treatments given alone, palonosetron and HM01 reduced retching and vomiting during the first 4-h period by 71.2% (*P* < 0.01) and 75.4% (*P* < 0.001), respectively; their combination produced 92.4% inhibition (*P* < 0.0001). Netupitant alone produced a reduction of only 43.4% (not statistically significant). When combined with palonosetron, the antagonism of emesis was 74.2% (*P* < 0.001); when combined with HM01, the reduction was 54.3%, but this result was not statistically significant (**Figure 8**). A similar level of inhibition was seen for the entire 24-h period. Thus, as treatments given alone, palonosetron and HM01 reduced retching and vomiting during the entire 24-h period by 62.5% (*P* < 0.01) and 73.3% (*P* < 0.001), respectively; their combination produced 83.6% inhibition (*P* < 0.0001). Netupitant alone produced a reduction of only 42.9% (not statistically significant). When combined with palonosetron, the antagonism of emesis was 66.1% (*P* < 0.001); when combined with HM01, the reduction was 45.2%, but this result was not statistically significant. The triple combination of palonosetron, netupitant and HM01 produced a significant 82.7% antagonism of emesis (*P* < 0.0001; **Figure 8**). None of the single treatments delayed the onset of the first episode of retching and/or vomiting (**Figure 8**). However, palonosetron combined with netupitant delayed the onset by 0.5 h (*P* < 0.05), palonosetron combined with HM01 delayed the onset by 3.1 h (*P* < 0.0001) and the triple combination of palonosetron, netupitant and HM01 delayed the onset by 0.8 h (*P* < 0.001). The control animals treated with cisplatin and vehicle lost 10.0% ± 0.9% of their starting body weight (**Figure 8**). During the observation period, the controls ate 30.0 ± 8.1 g/kg of food and consumed 91.3 ± 18.5 ml/kg of water (**Figure 8**). None of the treatments affected the amount of weight lost, the amount of food or water consumed, or the latencies to eat or drink (**Figure 8**).

## Discussion

This study is the first to report the unique anti-emetic spectrum of action of the orally bioavailable and brain-penetrating GHS-R1A mimetic, HM01, administered alone and/or in combination with standard anti-emetics such as palonosetron and netupitant. The complete inhibition by HM01 of the emetic response against cisplatin for the first 4 h was comparable with the efficacy of palonosetron (present data) and other 5-HT_3_ receptor antagonists that have been tested in *Suncus murinus* (Torii et al., 1991; Sam et al., 2003; Kwiatkowska et al., 2004; Tashiro et al., 2007; Ullah et al., 2017); a decrease in the number of retches and vomits and an increase in the latency to the first episode of emesis were seen. HM01 also antagonized motion-induced emesis, which distinguishes it from 5-HT_3_ receptor antagonists (Torii et al., 1991). In fact, the reduction of motion-induced emesis by HM01 in the model appears superior to the action of diphenhydramine and scopolamine (Tu et al., 2017), which are the gold-standard anti–motion sickness drugs used in humans (Golding and Gresty, 2015). HM01 did not antagonize emesis induced by nicotine or copper sulfate. This differentiates HM01 from the anti-emetic profile of the NK_1_ receptor antagonists, which reduce emesis induced by cisplatin, motion, nicotine and copper sulfate (Bountra et al., 1993; Gardner et al., 1995; Watson et al., 1995; Rudd et al., 1996). However, HM01 had positive effects on feeding and drinking after experiments involving nicotine and cisplatin, but not after that involving copper sulfate; the relevance of this finding is discussed below.

### HM01 Did Not Affect Emesis Induced by Nicotine or Copper Sulfate

The mechanism by which nicotine induces emesis is not entirely known and may involve an activation of multiple pathways. In dogs, nicotine-induced emesis is prevented by ablation of the area postrema and lesions of the vestibular apparatus; in cats, however, ablation of the area postrema is not effective, and experimentation suggests direct action in the brainstem and the involvement of autonomic ganglia and/or associated end organs in the periphery (Laffan and Borison, 1957). The pathways that mediate emesis induced by nicotine in *Suncus murinus* are not known, but the emesis is not blocked by 5-HT_3_ receptor antagonists (Torii et al., 1991). Oral administration of copper sulfate induces emesis mainly via the vagus and greater splanchnic nerves, but in contrast to the early emesis induced by cisplatin, the mechanism is not particularly affected by selective 5-HT_3_ receptor antagonists or by ablation of the area postrema (Wang and Borison, 1952; Costall et al., 1990; Torii et al., 1991). Whilst activation of the vagal afferents by copper sulfate can perturb emetic circuits to other challenges, it presumably occurs at the integrative level of the dorsal vagal complex and not necessarily via the vestibular system (Yates et al., 2014). Because the mechanisms and pathways activated by nicotine and copper sulfate are different from those activated by cisplatin and motion, it is not surprising that HM01 was ineffective against these stimuli.

### Anti-emetic Spectrum of Activity of HM01

Previous studies of the role of GHS-R1A in emesis control used a ferret model. Ghrelin administered by intraperitoneal injection (total dose, 1 mg/kg) produced a non-significant 20% reduction in the number of episodes of retching and/or vomiting (Rudd et al., 2006). However, when administered into the third ventricle of the brain, ghrelin (10–30 μg) reduced the number of episodes of retching and/or vomiting in a dose-dependent manner, with the maximum reduction reaching approximately 74% (Rudd et al., 2006). Whilst the anti-emetic action was impressive, it only lasted approximately 30 min, and it was reasoned that this transience was likely due to its relatively short half-life: the half-life in plasma is 30 min in rats and 9–13 min in humans (Tolle et al., 2002; Akamizu et al., 2004). In comparison, oral administration of HM01 reduced retching and vomiting by 80%, even during the longer 24-h observation period. The half-life of HM01 in *Suncus murinus* is not known, but it about 4.3 h in rats (Karasawa et al., 2014). Analysis of our data shows that HM01 more effectively antagonized emesis induced by cisplatin than that induced by motion. The ID_50_ value of HM01 against the emetic response to cisplatin during the first 4 h was approximately 6.0 mg/kg, and all animals were protected completely at 30 mg/kg. HM01 antagonized motion-induced emesis by approximately 77% at 10 mg/kg, but the effect was lost at 30 mg/kg.

Ghrelin levels have been reported to decrease during testing with an optokinetic stimulus in subjects with visually induced nausea and in patients who are undergoing chemotherapy (Matsumura et al., 2013; Farmer et al., 2015). In susceptible individuals, motion sickness is hypothesized to involve a mismatch of converging sensory information from the eyes, inner ears and proprioceptive input patterns that differ from learned and expected sensory pattern (Bertolini and Straumann, 2016). The mechanism by which motion induces emesis in *Suncus murinus* is not dependent on the abdominal vagi (Percie du Sert et al., 2010). Data from studies in *Suncus murinus* and in cats indicate that motion-induced emesis involves pathways in the brainstem nucleus tractus solitarius (NTS; likely to result from descending activation from vestibular input, not gastrointestinal input) and the lateral reticular formation, including the ventrolateral reticular formation, the inferior olive, the vestibular nuclei and the nucleus ambiguus (Ito et al., 2003, 2005; Balaban et al., 2014; Yates et al., 2014). Animals that have undergone labyrinthectomy and people with a defective labyrinth do not normally experience motion sickness or motion-induced emesis, but blind subjects have a normal motion-sickness response (Crampton, 1990). The emesis induced by our motion stimulus was probably driven mainly by the vestibular system, with afferents going to the vestibular nuclei. The early/acute emesis induced by cisplatin, in contrast, involves a peripheral drive of 5-HT (probably from enterochromaffin cells) via 5-HT_3_ receptors on vagal afferents and activation of the NTS and area postrema pathways (Rudd and Andrews, 2004). However, cisplatin is also known to cause vestibular ototoxicity (Rybak et al., 2009) and to increase c-fos in the brainstem (Chan et al., 2014), where signals from the vestibular nuclei eventually converge after motion (Yates et al., 2014). The crossover between the respective mechanisms by which cisplatin and motion induce emesis via hypothalamic, vestibular and medullary pathways interests us the most when we attempt to understand the anti-emetic mechanisms of action of HM01, because GHS-R1A is distributed in these brain areas (Bron et al., 2013) and because a history of motion sickness is a risk factor for chemotherapy-induced nausea and emesis in humans (Hesketh, 2008).

It is interesting that the effects of HM01 against motion-induced emesis were lost at the higher dose of 30 mg/kg. HM01 is highly selective for GHS-R1A, so off-target effects were not expected (Karasawa et al., 2014). Conversely, HM01 had a clear dose-response relationship to inhibit emesis induced by cisplatin. The difference in dose-response effects may therefore relate to GHS-R1A located in the different pathways modulated by motion and cisplatin. Certainly, it was noted in the original ferret experiments that although ghrelin was anti-emetic, it did appear to induce a few episodes of emesis shortly after intracerebroventricular administration (Rudd et al., 2006). In our experiments, HM01 was administered orally and would have been gradually absorbed rather than quickly presenting in the brain at a high concentration (Karasawa et al., 2014). We saw no evidence that HM01 itself could induce emesis or could potentiate emesis to any of the emetic challenges that we studied. It is therefore unknown why HM01 was less effective against motion-induced emesis when used at the higher dose (30 mg/kg).

### Feeding, Drinking and Weight Loss

It is known that stimulation of GHS-R1A in the brainstem and hypothalamus are involved in appetite mechanisms, with driving input also involving GHS-R1A on the vagus nerve via the NTS (Olszewski et al., 2003). It is also known that injection of ghrelin into the amygdala inhibits conditioned taste aversion induced by lithium chloride (Alvarez-Crespo et al., 2012). The hypothalamus, amygdala and brainstem, including the area postrema and the NTS, are involved in the emetic mechanism of action of cisplatin (Rudd and Andrews, 2004; De Jonghe and Horn, 2009) and in the mechanisms of action of conditioned taste aversion and pica in rats (Parker, 2014). It is possible, therefore, that HM01 might have reduced ‘nausea’ and stimulated feeding during the experiments with cisplatin and perhaps other emetic challenges. We expected that animals protected from ‘nausea’ and emesis would resume feeding and drinking more quickly than the control animals. However, our experiments were primarily designed and powered to record emesis. To reduce the number of animals used, we did not use vehicle control groups for the emetogens. We were interested to find, however, that the baseline food consumption of animals that had received nicotine and copper sulfate differed markedly: animals that received nicotine, copper sulfate and cisplatin ate approximately 3.5, 0.4, and 29.5 g/kg food (24-h recording), respectively; a different pattern was seen in water consumption: 10.3, 20.2, and 146.1 ml/kg (24-h recording), respectively.

In our laboratory, normal untreated adult male *Suncus murinus* eat approximately 100 g/kg food and consume approximately 267 ml/kg of water each day, which agrees with published values (Andrews et al., 2005). It is likely, therefore, that the three emetic challenges reduced food and water consumption, but by varying degrees. Nevertheless, HM01 only actively increased food and water consumption in the nicotine-treated animals, and this was accompanied by a reduction in weight loss, without affecting the latencies to eat or drink. The relevance of the data is therefore difficult to rationalize to mechanisms of nausea and emesis. However, whilst HM01 did not affect the amounts of food and water consumed in cisplatin-treated animals, it did shorten their latency to drink. These observations must be treated cautiously, but they tend to agree with the view that stimulation of feeding may not necessarily inhibit emetic pathways (Horn et al., 2010). Yet we must be cautious in making such assumptions, as other experiments in rats show that the effect of HM01 (dosed orally once per day at 3 and 10 mg/kg) to increase food and water intake requires 1 week to develop (Karasawa et al., 2014) and that the effect manifests more quickly if HM01 is delivered intravenously by osmotic minipumps (Karasawa et al., 2014). It may be that treatment with HM01 must begin several days before the emetic challenge to fully realize any beneficial effect. Although the literature contains no information to conclude that 5-HT_3_ or NK_1_ receptors antagonists can stimulate feeding, both drug classes reduce nausea and vomiting in chemotherapy patients (Rudd and Andrews, 2004). However, neither palonosetron nor netupitant showed activity to modify the latencies to eat or drink or to affect food and water consumption and body weight loss in animals treated with cisplatin. This finding may indicate that although retching and vomiting was reduced, gastric malaise and/or ‘nausea’ (if experienced at all), persists via mechanisms that are not regulated by the 5-HT_3_ or NK_1_ receptors.

### Usefulness of HM01 in Combination With Palonosetron and/or Netupitant

Palonosetron is a ‘second-generation’ 5-HT_3_ receptor antagonist that is approximately ten times more potent than older compounds at blocking 5-HT_3_ receptor and whose plasma half-life is approximately three times longer (Wong et al., 1995; Grunberg and Koeller, 2003). A previous study in *Suncus murinus* that did not assess emetic events reported that palonosetron (0.5 mg/kg by intraperitoneal injection) reduced cisplatin-induced (30 mg/kg by intraperitoneal injection) c-fos increase in the NTS, the dorsal motor nucleus of the vagus nerve and the central nucleus of the amygdala at 6 h; these protective effects were not seen in any brain areas when compared at 48 h (De Jonghe and Horn, 2009). In a second study, palonosetron 0.5 mg/kg by subcutaneous injection reduced the number of emetic episodes over the first 24-h period by 87.9% (Ullah et al., 2017); this finding is highly comparable with the 84.9% inhibition seen after oral administration of 1 mg/kg palonosetron in the present study. Indeed, palonosetron had an ID_50_ of approximately 0.0098 mg/kg, which is similar to its potency in ferrets and dogs in acute experimentation of cisplatin, in which the ID_50_ values were 0.003 and 0.008 mg/kg, respectively (Eglen et al., 1995). Netupitant (0.3 mg/kg, p.o.) potently reduces cisplatin-induced acute emesis in ferrets by approximately 95%, with an estimated IC_50_ value of 0.1 mg/kg, p.o. (Rudd et al., 2016). Yet in this present study, netupitant was relatively less potent in reducing cisplatin-induced emesis; a reduction of only around 40% was seen at 3 mg/kg. However, a previous study in *Suncus murinus* found that netupitant could antagonize motion-mediated emesis with an ID_50_ value of 0.08 mg/kg, p.o. (Rudd et al., 2016). In ferrets, netupitant is known to have a long duration of action of approximately 96 h (Rudd et al., 2016). If netupitant also has a long half-life in *Suncus murinus*, the difference in potency against emesis induced by cisplatin and by motion is more likely to relate to the relative role of substance P in emetic mechanisms than to its pharmacokinetic profile. In contrast, NK_1_ antagonists are more effective in treating the delayed phase associated with high emetogenic chemotherapy, whereas in this study, our investigation modeled the acute phase more closely. It appears that the pattern of activity of netupitant against motion and cisplatin is shared with other NK_1_ receptor antagonists. Thus, GR203040 (3 mg/kg by subcutaneous injection) produces 77.5 and 53.0% reduction of emesis induced by motion and cisplatin, respectively (Gardner et al., 1996); another NK_1_-selective antagonist, GR205171 (3 mg/kg by subcutaneous injection), produced 91.2 and 57.2% reductions in emesis induced by motion and cisplatin, respectively (Gardner et al., 1996); and CP-99944 (10 mg/kg by subcutaneous injection) prevented motion-induced emesis completely (Rudd et al., 1999) but only reduced cisplatin-induced emesis by 40.7% (Lau et al., 2005). This tentatively suggests that the levels of substance P and/or other mediators in emetic circuits may be higher after administration of cisplatin than after motion (Andrews and Rudd, 2004).

Clearly, the mechanism of action by which HM01 antagonizes emesis differs qualitatively and quantitatively from those of palonosetron and netupitant. We therefore designed experiments to examine the effects of HM01 when combined with suboptimal doses of palonosetron and netupitant. At the doses used alone, none of the treatments could effectively increase the latency to the first emetic episode. However, the combinations of palonosetron plus netupitant, palonosetron plus HM01, and palonosetron plus netupitant and HM01 increased the latency to the first episode. It was not unexpected that palonosetron and netupitant would have useful effects on latency when combined because a similar positive interaction has been observed in ferrets and in humans (Navari, 2015; Rudd et al., 2016). Ghrelin has been shown to have beneficial effects against chemotherapy-induced nausea in patients who concurrently receive treatment with the 5-HT_3_ receptor antagonist, ramosetron (Hiura et al., 2012). In the former study, however, the effects of ghrelin alone were not determined. Our study showed that HM01 had useful effects to increase the latency to the first episode of retching and/or vomiting when used alone (dose ranging study) and when a suboptimal dose was combined with palonosetron, but not with netupitant.

Although we observed a positive interaction between palonosetron and netupitant that increased the latency to the first episode of cisplatin-induced retching and/or vomiting, no interaction was seen to reduce the actual numbers of retches and/or vomits. This differs from clinical observations, in which the combination of palonosetron plus netupitant is better than palonosetron alone against acute and delayed nausea and vomiting induced by moderately emetogenic chemotherapy (Lorusso et al., 2015); a positive interaction to reduce retching and vomiting induced by cisplatin has also been seen in the least shrew when assessed over a 16-h period (Darmani et al., 2015). *Suncus murinus* tachykinin NK_1_ receptors are not rodent- or human-like (Andrews and Rudd, 2004), but our unpublished studies on *Suncus murinus* isolated ileum revealed that netupitant has a pKb of 8.3 to antagonize [Sar^9^Met(O_2_)^11^]–substance P–induced contractions (**Supplementary Figure S1**); this is similar to its potency against substance P in guinea pig isolated ileum (pKb = 8.87), but slightly less than expected from binding data on human NK_1_ receptors expressed in CHO cells (pKi = 9) (Rizzi et al., 2012). In contrast, our unpublished data show that palonosetron non-competitively antagonizes 2-methyl-5-HT–induced contractions of *Suncus murinus* isolated ileum, with a pKb of 11 (**Supplementary Figure S2**), which is approximately 100 times more potent than in guinea pig ileum to also reduce 2-methyl-5-HT induced contractions (pKb = 8.8) and 10 times more potent compared with binding studies on human hippocampal tissues that express 5-HT_3_ receptors (pKi = 10) (Wong et al., 1995). Palonosetron can cause 5-HT_3_ receptor internalization and has unique actions to reduce cross-talk between NK_1_ and 5-HT_3_ receptor signaling pathways, which goes some way to explaining its enhanced efficacy against cisplatin-induced delayed emesis (Rojas et al., 2010). The acute nature of our cisplatin-induced emesis experiments may explain why we were unable to detect this interaction. Interestingly, it has been reported that GHS-R1A can dimerize with melanocortin-3, dopamine (D_1_ and D_2_) and 5-HT_2C_ receptors (Schellekens et al., 2013). Several of these receptors are involved in emetic mechanisms, but there is no evidence to date that GHS-R1A interacts directly with 5-HT_3_ or NK_1_ receptors.

## Conclusion

HM01 was revealed to have useful anti-emetic properties against the chemotherapeutic drug, cisplatin. The anti-emetic effect over the first 4 h appeared comparable with the efficacy of palonosetron and other 5-HT_3_ receptor antagonists previously tested in *Suncus murinus*. Moreover, HM01 also reduced motion-induced emesis, thus revealing for the first time that brain-penetrating GHS-R1A agonists may have clinical utility in the treatment of motion sickness in humans. The link between motion sickness susceptibility and chemotherapy-induced nausea and emesis is well known (Shih et al., 2009; Bouganim et al., 2012). We have shown that HM01 was useful alone, in combination with palonosetron and in combination with a palonosetron and netupitant regimen for the control of chemotherapy-induced (acute) emesis; however, it was less useful against emesis induced by pathways modulated by nicotine and intragastric copper sulfate. Given the link between motion sickness susceptibility and post-operative nausea and vomiting (Lee et al., 2007) and pregnancy sickness (Mullin et al., 2012), it is tempting to speculate that GHS-R1A agonists may have greater clinical utility in reducing the side effects of nausea and vomiting in other areas of medicine in which effective and safe drugs are lacking.

## Author Contributions

JR, CG, CP, EL, and LT designed the research study. MN, LT, and ZL performed the study. JR, SC, LT, and ZL analyzed the data. JR, LT, ZL, and CP wrote the manuscript.

## Declaration of Transparency and Scientific Rigor

This declaration acknowledges that this paper adheres to the principles for transparent reporting and scientific rigor of preclinical research recommended by funding agencies, publishers and other organizations engaged with supporting research.

## Conflict of Interest Statement

The research has been partly funded by an Helsinn grant. CG, EL, and CP are Helsinn employers. The remaining authors declare that the research was conducted in the absence of any commercial or financial relationships that could be construed as a potential conflict of interest.

## Supplementary Material

FIGURE S1 Effect of netupitant on [Sar^9^Met(O_2_)^11^]-substance P -induced contractions of *Suncus murinus* isolated ileum **(top)**; double reciprocal plot for [Sar^9^Met(O_2_)^11^]-substance P in the presence of 30 nM netupitant **(bottom)**. Data represents the mean ± SEM of 6 determinations.

FIGURE S2 Effect of palonosetron on 2-methyl-5-HT-induced contractions of *Suncus murinu*s isolated ileum **(top)**; double reciprocal plot for 2-methyl-5-HT in the presence of 0.03 nM palonosetron **(bottom)**. Data represents the mean ± SEM of 6 determinations.

## References

[B1] Akamizu T. Takaya K. Irako T. Hosoda H. Teramukai S. Matsuyama A. (2004). Pharmacokinetics, safety, and endocrine and appetite effects of ghrelin administration in young healthy subjects. *Eur. J. Endocrinol.* 150 447–455. 10.1530/eje.0.1500447 15080773

[B2] Alvarez-Crespo M. Skibicka K. P. Farkas I. Molnar C. S. Egecioglu E. Hrabovszky E. (2012). The amygdala as a neurobiological target for ghrelin in rats: neuroanatomical, electrophysiological and behavioral evidence. *PLoS One* 7:e46321. 10.1371/journal.pone.0046321 23071554 PMC3468604

[B3] Andrews P. L. R. Rudd J. A. (2004). “The role of tachykinins and the tachykinin receptor in nausea and emesis,” in *Handbook of Experimental Pharmacology*, ed. Holzer P. (Berlin, Germany: Springer-Verlag Berlin Heidelberg), 359–440.

[B4] Andrews P. L. R. Rudd J. A. (2015). “The physiology and pharmacology of nausea and vomiting induced by anti-cancer chemotherapy in humans,” in *Management of Chemotherapy-Induced Nausea and Vomiting: New Agents and New Uses of Current Agents*, ed. Navari R. (London: Springer Health Care Publishers).

[B5] Andrews P. L. R. Friedman M. I. Liu Y. L. Smith J. E. Sims D. W. (2005). Potential energetic implications of emesis in the house musk shrew (*Suncus murinus*). *Physiol. Behav.* 84 519–524. 10.1016/j.physbeh.2005.01.010 15811386

[B6] Balaban C. D. Ogburn S. W. Warshafsky S. G. Ahmed A. Yates B. J. (2014). Identification of neural networks that contribute to motion sickness through principal components analysis of fos labeling induced by galvanic vestibular stimulation. *PLoS One* 9:e86730. 10.1371/journal.pone.0086730 24466215 PMC3900607

[B7] Beleslin D. B. Krstic S. K. (1987). Further studies on nicotine-induced emesis: nicotinic mediation in area postrema. *Physiol. Behav.* 39 681–686. 10.1016/0031-9384(87)90250-2 3602120

[B8] Bertolini G. Straumann D. (2016). Moving in a moving world: a review on vestibular motion sickness. *Front Neurol* 7:14. 10.3389/fneur.2016.00014 26913019 PMC4753518

[B9] Bouganim N. Dranitsaris G. Hopkins S. Vandermeer L. Godbout L. Dent S. (2012). Prospective validation of risk prediction indexes for acute and delayed chemotherapy-induced nausea and vomiting. *Curr. Oncol.* 19 e414–e421. 10.3747/co.19.1074 23300365 PMC3503672

[B10] Bountra C. Bunce K. T. Dale T. Gardner C. Jordan C. Twissell D. (1993). Anti-emetic profile of a non-peptide neurokinin NK1 receptor antagonist, CP-99,994, in ferrets. *Eur. J. Pharmacol* 249 R3–R4. 10.1016/0014-2999(93)90673-6 7506663

[B11] Bron R. Yin L. Russo D. Furness J. B. (2013). Expression of the ghrelin receptor gene in neurons of the medulla oblongata of the rat. *J. Comp. Neurol.* 521 2680–2702. 10.1002/cne.23309 23348715

[B12] Camilleri M. Acosta A. (2015). Emerging treatments in neurogastroenterology: relamorelin: a novel gastrocolokinetic synthetic ghrelin agonist. *Neurogastroenterol. Motil.* 27 324–332. 10.1111/nmo.12490 25545036 PMC4424792

[B13] Chan S. W. Lu Z. Lin G. Yew D. T. Yeung C. K. Rudd J. A. (2014). The differential antiemetic properties of GLP-1 receptor antagonist, exendin (9-39) in *Suncus murinus* (house musk shrew). *Neuropharmacology* 83 71–78. 10.1016/j.neuropharm.2014.03.016 24726308

[B14] Costall B. Domeney A. M. Naylor R. J. Owera-Atepo J. B. Rudd J. A. Tattersall F. D. (1990). Fluphenazine, ICS 205-930 and dl-fenfluramine differentially antagonise drug-induced emesis in the ferret. *Neuropharmacology* 29 453–462. 10.1016/0028-3908(90)90167-P 1972549

[B15] Crampton G.H. (1990). “Neurophysiology of motion sickness,” in *Motion and Space Sickness*, ed. Crampton G. H. (Boca Raton, FL: CRC Press Inc.), 29–42.

[B16] Currow D. C. Abernethy A. P. (2014). Anamorelin hydrochloride in the treatment of cancer anorexia-cachexia syndrome. *Future Oncol* 10 789–802. 10.2217/fon.14.14 24472001

[B17] Darmani N. A. Zhong W. Chebolu S. Mercadante F. (2015). Differential and additive suppressive effects of 5-HT3 (palonosetron)- and NK1 (netupitant)-receptor antagonists on cisplatin-induced vomiting and ERK1/2, PKA and PKC activation. *Pharmacol. Biochem. Behav.* 131 104–111. 10.1016/j.pbb.2015.02.010 25687374

[B18] De Jonghe B. C. Horn C. C. (2009). Chemotherapy agent cisplatin induces 48-h Fos expression in the brain of a vomiting species, the house musk shrew (*Suncus murinus*). *Am. J. Physiol. Regul. Integr. Comp. Physiol.* 296 R902–R911. 10.1152/ajpregu.90952.2008 19225146 PMC2698611

[B19] Eglen R. M. Lee C. H. Smith W. L. Johnson L. G. Clark R. Whiting R. L. (1995). Pharmacological characterization of RS 25259-197, a novel and selective 5-HT3 receptor antagonist, in vivo. *Br. J. Pharmacol.* 114 860–866. 10.1111/j.1476-5381.1995.tb13283.x 7773547 PMC1510198

[B20] Farmer A. D. Ban V. F. Coen S. J. Sanger G. J. Barker G. J. Gresty M. A. (2015). Visually induced nausea causes characteristic changes in cerebral, autonomic and endocrine function in humans. *J. Physiol.* 593 1183–1196. 10.1113/jphysiol.2014.284240 25557265 PMC4358679

[B21] Gardner C. J. Armour D. R. Beattie D. T. Gale J. D. Hawcock A. B. Kilpatrick G. J. (1996). GR205171: a novel antagonist with high affinity for the tachykinin NK1 receptor, and potent broad-spectrum anti-emetic activity. *Regul Pept.* 65 45–53. 10.1016/0167-0115(96)00071-7 8876035

[B22] Gardner C. J. Twissell D. J. Dale T. J. Gale J. D. Jordan C. C. Kilpatrick G. J. (1995). The broad-spectrum anti-emetic activity of the novel non-peptide tachykinin NK1 receptor antagonist GR203040. *Br. J. Pharmacol.* 116 3158–3163. 10.1111/j.1476-5381.1995.tb15118.x 8719790 PMC1909155

[B23] Golding J. F. Gresty M. A. (2015). Pathophysiology and treatment of motion sickness. *Curr. Opin. Neurol* 28 83–88. 10.1097/WCO.0000000000000163 25502048

[B24] Gralla R. J. Bosnjak S. M. Hontsa A. Balser C. Rizzi G. Rossi G. (2014). A phase III study evaluating the safety and efficacy of NEPA, a fixed-dose combination of netupitant and palonosetron, for prevention of chemotherapy-induced nausea and vomiting over repeated cycles of chemotherapy. *Ann. Oncol.* 25 1333–1339. 10.1093/annonc/mdu096 24631949 PMC4071753

[B25] Grunberg S. M. Koeller J. M. (2003). Palonosetron: a unique 5-HT3-receptor antagonist for the prevention of chemotherapy-induced emesis. *Expert Opin. Pharmacother.* 4 2297–2303. 10.1517/14656566.4.12.2297 14640928

[B26] Hesketh P. J. (2008). Chemotherapy-induced nausea and vomiting. *N. Engl. J. Med.* 358 2482–2494. 10.1056/NEJMra0706547 18525044

[B27] Hesketh P. J. Rossi G. Rizzi G. Palmas M. Alyasova A. Bondarenko I. (2014). Efficacy and safety of NEPA, an oral combination of netupitant and palonosetron, for prevention of chemotherapy-induced nausea and vomiting following highly emetogenic chemotherapy: a randomized dose-ranging pivotal study. *Ann. Oncol.* 25 1340–1346. 10.1093/annonc/mdu110 24608196 PMC4071755

[B28] Hiura Y. Takiguchi S. Yamamoto K. Takahashi T. Kurokawa Y. Yamasaki M. (2012). Effects of ghrelin administration during chemotherapy with advanced esophageal cancer patients: a prospective, randomized, placebo-controlled phase 2 study. *Cancer* 118 4785–4794. 10.1002/cncr.27430 22282373

[B29] Horn C. C. Still L. Fitzgerald C. Friedman M. I. (2010). Food restriction, refeeding, and gastric fill fail to affect emesis in musk shrews. *Am. J. Physiol. Gastrointest. Liver Physiol.* 298 G25–G30. 10.1152/ajpgi.00366.2009 19892939 PMC2806101

[B30] Ishida Y. Sakahara S. Tsutsui C. Kaiya H. Sakata I. Oda S. (2009). Identification of ghrelin in the house musk shrew (*Suncus murinus*): cDNA cloning, peptide purification and tissue distribution. *Peptides* 30 982–990. 10.1016/j.peptides.2009.01.006 19428777

[B31] Ito H. Nishibayashi M. Kawabata K. Maeda S. Seki M. Ebukuro S. (2003). Induction of Fos protein in neurons in the medulla oblongata after motion- and X-irradiation-induced emesis in musk shrews (*Suncus murinus*). *Auton. Neurosci.* 107 1–8. 10.1016/S1566-0702(03)00026-2 12927221

[B32] Ito H. Nishibayashi M. Maeda S. Seki M. Ebukuro S. (2005). Emetic responses and neural activity in young musk shrews during the breast-feeding/weaning period: comparison between the high and low emetic response strains using a shaking stimulus. *Exp. Anim.* 54 301–307. 10.1538/expanim.54.301 16093643

[B33] Kan K. K. Rudd J. A. Wai M. K. (2006). Differential action of anti-emetic drugs on defecation and emesis induced by prostaglandin E2 in the ferret. *Eur. J. Pharmacol* 544 153–159. 10.1016/j.ejphar.2006.06.034 16844111

[B34] Karasawa H. Pietra C. Giuliano C. Garcia-Rubio S. Xu X. Yakabi S. (2014). New ghrelin agonist, HM01 alleviates constipation and L-dopa-delayed gastric emptying in 6-hydroxydopamine rat model of Parkinson’s disease. *Neurogastroenterol. Motil.* 26 1771–1782. 10.1111/nmo.12459 25327342 PMC4457321

[B35] Kwiatkowska M. Parker L. A. Burton P. Mechoulam R. (2004). A comparative analysis of the potential of cannabinoids and ondansetron to suppress cisplatin-induced emesis in the *Suncus murinus* (house musk shrew). *Psychopharmacology (Berl.)* 174 254–259. 10.1111/nmo.12459 14740147

[B36] Laffan R. J. Borison H. L. (1957). Emetic action of nicotine and lobeline. *J. Pharmacol. Exp. Ther.* 121 468–476. 13492162

[B37] Lau A. H. Rudd J. A. Yew D. T. (2005). Action of ondansetron and CP-99,994 on cisplatin-induced emesis and locomotor activity in *Suncus murinus* (house musk shrew). *Behav. Pharmacol.* 16 605–612. 10.1097/00008877-200512000-00002 16286811

[B38] Lee Y. Y. Kim K. H. Yom Y. H. (2007). Predictive models for post-operative nausea and vomiting in patients using patient-controlled analgesia. *J. Int. Med. Res.* 35 497–507. 10.1177/147323000703500409 17697527

[B39] Lorusso V. Karthaus M. Aapro M. (2015). Review of oral fixed-dose combination netupitant and palonosetron (NEPA) for the treatment of chemotherapy-induced nausea and vomiting. *Future Oncol.* 11 565–577. 10.2217/fon.14.260 25360998

[B40] Matsumura T. Arai M. Yoshikawa M. Sudo K. Nakamura K. Katsuno T. (2013). Changes in plasma ghrelin and serum leptin levels after Cisplatin-based transcatheter arterial infusion chemotherapy for hepatocellular carcinoma. *ISRN Gastroenterol.* 2013:415450. 10.1155/2013/415450 23533792 PMC3606724

[B41] Miyano Y. Sakata I. Kuroda K. Aizawa S. Tanaka T. Jogahara T. (2013). The role of the vagus nerve in the migrating motor complex and ghrelin- and motilin-induced gastric contraction in suncus. *PLoS One* 8:e64777. 10.1371/journal.pone.0064777 23724093 PMC3665597

[B42] Mondal A. Aizawa S. Sakata I. Goswami C. Oda S. Sakai T. (2013). Mechanism of ghrelin-induced gastric contractions in *Suncus murinus* (house musk shrew): involvement of intrinsic primary afferent neurons. *PLoS One* 8:e60365. 10.1371/journal.pone.0060365 23565235 PMC3614873

[B43] Muller T. D. Nogueiras R. Andermann M. L. Andrews Z. B. Anker S. D. Argente J. (2015). Ghrelin. *Mol Metab* 4 437–460. 10.1016/j.molmet.2015.03.005 26042199 PMC4443295

[B44] Mullin P. M. Ching C. Schoenberg F. Macgibbon K. Romero R. Goodwin T. M. (2012). Risk factors, treatments, and outcomes associated with prolonged hyperemesis gravidarum. *J. Matern. Fetal Neonatal Med.* 25 632–636. 10.3109/14767058.2011.598588 21916750 PMC3560915

[B45] Navari R. M. (2015). Profile of netupitant/palonosetron (NEPA) fixed dose combination and its potential in the treatment of chemotherapy-induced nausea and vomiting (CINV). *Drug design, development and therapy* 9 155–161. 10.2147/DDDT.S76158 25552904 PMC4277122

[B46] Ogawa A. Mochiki E. Yanai M. Morita H. Toyomasu Y. Ogata K. (2012). Interdigestive migrating contractions are coregulated by ghrelin and motilin in conscious dogs. *Am. J. Physiol. Regul. Integr. Comp. Physiol.* 302 R233–R241. 10.1152/ajpregu.00078.2011 22071157

[B47] Olszewski P. K. Grace M. K. Billington C. J. Levine A. S. (2003). Hypothalamic paraventricular injections of ghrelin: effect on feeding and c-Fos immunoreactivity. *Peptides* 24 919–923. 10.1016/S0196-9781(03)00159-1 12948845

[B48] Oman C. M. (2012). Are evolutionary hypotheses for motion sickness “just-so” stories? J Vestib Res 22 117–127. 10.3233/VES-2011-0432 23000611

[B49] Parker L. A. (2014). Conditioned flavor avoidance and conditioned gaping: rat models of conditioned nausea. *Eur. J. Pharmacol.* 722 122–133. 10.1016/j.ejphar.2013.09.070 24157975

[B50] Percie du Sert N. Chu K. M. Wai M. K. Rudd J. A. Andrews P. L. (2010). Telemetry in a motion-sickness model implicates the abdominal vagus in motion-induced gastric dysrhythmia. *Exp. Physiol.* 95 768–773. 10.1113/expphysiol.2009.052001 20360423

[B51] Rizzi A. Campi B. Camarda V. Molinari S. Cantoreggi S. Regoli D. (2012). In vitro and in vivo pharmacological characterization of the novel NK1 receptor selective antagonist Netupitant. *Peptides* 37 86–97. 10.1016/j.peptides.2015.03.021 22732666

[B52] Roila F. Warr D. Hesketh P. J. Gralla R. Herrstedt J. Jordan K. (2017). 2016 updated MASCC/ESMO consensus recommendations: prevention of nausea and vomiting following moderately emetogenic chemotherapy. *Support Care Cancer* 25 289–294. 10.1007/s00520-016-3365-1 27510316

[B53] Rojas C. Li Y. Zhang J. Stathis M. Alt J. Thomas A. G. (2010). The antiemetic 5-HT3 receptor antagonist Palonosetron inhibits substance P-mediated responses in vitro and in vivo. *J. Pharmacol. Exp. Ther.* 335 362–368. 10.1124/jpet.110.166181 20724484 PMC3202469

[B54] Rudd J. A. Andrews P. L. R. (2004). “Mechanisms of acute, delayed and anticipatory vomiting in cancer and cancer treatment,” in *Management of Nausea and Vomiting in Cancer and Cancer Treatment*, ed. Hesketh P. (New York, NY: Jones and Barlett Publishers Inc.), 15–66.

[B55] Rudd J. A. Jordan C. C. Naylor R. J. (1996). The action of the NK1 tachykinin receptor antagonist, CP 99,994, in antagonizing the acute and delayed emesis induced by cisplatin in the ferret. *Br. J. Pharmacol.* 119 931–936. 10.1111/j.1476-5381.1996.tb15761.x 8922742 PMC1915933

[B56] Rudd J. A. Ngan M. P. Wai M. K. (1999). Inhibition of emesis by tachykinin NK1 receptor antagonists in *Suncus murinus* (house musk shrew). *Eur. J. Pharmacol.* 366 243–252. 10.1016/S0014-2999(98)00920-0 10082206

[B57] Rudd J. A. Ngan M. P. Lu Z. Higgins G. A. Giuliano C. Lovati E. (2016). Profile of antiemetic activity of netupitant alone or in combination with palonosetron and dexamethasone in ferrets and *Suncus murinus* (house musk shrew). *Front Pharmacol.* 7:263. 10.3389/fphar.2016.00263 27630563 PMC5005416

[B58] Rudd J. A. Ngan M. P. Wai M. K. King A. G. Witherington J. Andrews P. L. R. (2006). Anti-emetic activity of ghrelin in ferrets exposed to the cytotoxic anti-cancer agent cisplatin. *Neurosci. Lett.* 392 79–83. 10.1016/j.neulet.2005.08.062 16182445

[B59] Rybak L. P. Mukherjea D. Jajoo S. Ramkumar V. (2009). Cisplatin ototoxicity and protection: clinical and experimental studies. *Tohoku J. Exp. Med.* 219 177–186. 10.1620/tjem.219.177 19851045 PMC2927105

[B60] Sam T. S. Cheng J. T. Johnston K. D. Kan K. K. Ngan M. P. Rudd J. A. (2003). Action of 5-HT3 receptor antagonists and dexamethasone to modify cisplatin-induced emesis in *Suncus murinus* (house musk shrew). *Eur. J. Pharmacol.* 472 135–145. 10.1016/S0014-2999(03)01863-6 12860482

[B61] Sanger G. J. Broad J. Andrews P. L. R. (2013). The relationship between gastric motility and nausea: gastric prokinetic agents as treatments. *Eur. J. Pharmacol.* 715 10–14. 10.1016/j.ejphar.2013.06.031 23831391

[B62] Schellekens H. Van Oeffelen W. E. Dinan T. G. Cryan J. F. (2013). Promiscuous dimerization of the growth hormone secretagogue receptor (GHS-R1a) attenuates ghrelin-mediated signaling. *J. Biol. Chem.* 288 181–191. 10.1074/jbc.M112.382473 23161547 PMC3537012

[B63] Shaw M. Pediconi C. Mcvey D. Mondou E. Quinn J. Chamblin B. (2013). Safety and efficacy of ulimorelin administered postoperatively to accelerate recovery of gastrointestinal motility following partial bowel resection: results of two randomized, placebo-controlled phase 3 trials. *Dis. Colon. Rectum.* 56 888–897. 10.1097/DCR.0b013e31829196d0 23739196

[B64] Shih V. Wan H. S. Chan A. (2009). Clinical predictors of chemotherapy-induced nausea and vomiting in breast cancer patients receiving adjuvant doxorubicin and cyclophosphamide. *Ann. Pharmacother.* 43 444–452. 10.1345/aph.1L437 19193584

[B65] Suzuki A. Ishida Y. Aizawa S. Sakata I. Tsutsui C. Mondal A. (2012). Molecular identification of GHS-R and GPR38 in *Suncus murinus*. *Peptides* 36 29–38. 10.1016/j.peptides.2012.04.019 22579813

[B66] Tashiro N. Kataoka M. Ozawa K. Ikeda T. (2007). The evaluation of whole-body plethysmography as a semiautomated method for analysis of emesis in the house musk shrew (*Suncus murinus*). *J. Am. Assoc. Lab. Anim. Sci.* 46 81–85. 17343358

[B67] Tolle V. Bassant M. H. Zizzari P. Poindessous-Jazat F. Tomasetto C. Epelbaum J. (2002). Ultradian rhythmicity of ghrelin secretion in relation with GH, feeding behavior, and sleep-wake patterns in rats. *Endocrinology* 143 1353–1361. 10.1210/endo.143.4.8712 11897692

[B68] Torii Y. Saito H. Matsuki N. (1991). Selective blockade of cytotoxic drug-induced emesis by 5-HT3 receptor antagonists in *Suncus murinus*. *Jpn. J. Pharmacol.* 55 107–113. 10.1254/jjp.55.107 2041220

[B69] Tu L. Lu Z. Dieser K. Schmitt C. Chan S. W. Ngan M. P. (2017). Brain activation by H1 antihistamines challenges conventional view of their mechanism of action in motion sickness: a behavioral, c-fos and physiological study in *Suncus murinus* (House Musk Shrew). *Front. Physiol.* 8:412. 10.3389/fphys.2017.00412 28659825 PMC5470052

[B70] Ullah I. Subhan F. Lu Z. Chan S. W. Rudd J. A. (2017). Action of Bacopa monnieri to antagonize cisplatin-induced emesis in *Suncus murinus* (house musk shrew). *J. Pharmacol. Sci.* 133 232–239. 10.1016/j.jphs.2017.03.001 28363413

[B71] Van den Brande J. Brouwer A. Peeters M. (2014). Use of antiemetics in the prevention of chemotherapy-induced nausea and vomiting: review and focus on the Belgian situation. *Acta Gastroenterol. Belg.* 77 240–248. 25090823

[B72] Villars F. O. Pietra C. Giuliano C. Lutz T. A. Riediger T. (2017). Oral Treatment with the ghrelin receptor agonist HM01 attenuates cachexia in mice bearing colon-26 (C26) tumors. *Int. J. Mol. Sci.* 18:E986. 10.3390/ijms18050986 28475119 PMC5454899

[B73] Wang S. C. Borison H. L. (1952). A new concept of organization of the central emetic mechanism: recent studies on the sites of action of apomorphine, copper sulfate and cardiac glycosides. *Gastroenterology* 22 1–12. 12980223

[B74] Watson J. W. Gonsalves S. F. Fossa A. A. Mclean S. Seeger T. Obach S. (1995). The anti-emetic effects of CP-99,994 in the ferret and the dog: role of the NK1 receptor. *Br. J. Pharmacol.* 115 84–94. 10.1111/j.1476-5381.1995.tb16324.x 7544198 PMC1908747

[B75] Wong E. H. Clark R. Leung E. Loury D. Bonhaus D. W. Jakeman L. (1995). The interaction of RS 25259-197, a potent and selective antagonist, with 5-HT3 receptors, in vitro. *Br. J. Pharmacol.* 114 851–859. 10.1111/j.1476-5381.1995.tb13282.x 7773546 PMC1510197

[B76] Yates B. J. Catanzaro M. F. Miller D. J. Mccall A. A. (2014). Integration of vestibular and emetic gastrointestinal signals that produce nausea and vomiting: potential contributions to motion sickness. *Exp. Brain Res.* 232 2455–2469. 10.1007/s00221-014-3937-6 24736862 PMC4112154

