# Corrigendum: Anti-emetic Action of the Brain-Penetrating New Ghrelin Agonist, HM01, Alone and in Combination With the 5-HT_3_ Antagonist, Palonosetron and With the NK_1_ Antagonist, Netupitant, Against Cisplatin- and Motion-Induced Emesis in *Suncus murinus* (House Musk Shrew)

**DOI:** 10.3389/fphar.2018.01102

**Published:** 2018-09-28

**Authors:** John A. Rudd, Sze W. Chan, Man P. Ngan, Longlong Tu, Zengbing Lu, Claudio Giuliano, Emanuela Lovati, Claudio Pietra

**Affiliations:** ^1^Emesis Research Group, School of Biomedical Sciences, Faculty of Medicine, The Chinese University of Hong Kong, Shatin, Hong Kong; ^2^Brain and Mind Institute, The Chinese University of Hong Kong, Shatin, Hong Kong; ^3^School of Health Sciences, Caritas Institute of Higher Education, Tseung Kwan O New Town, Hong Kong; ^4^Helsinn Healthcare SA, Research and Development, Lugano, Switzerland

**Keywords:** ghrelin, HM01, nausea, emesis, chemotherapy, motion

An author name was incorrectly spelled as Zhengbing Lu. The correct spelling is Zengbing Lu. The authors apologize for this error and state that this does not change the scientific conclusions of the article in any way.

The original article has been updated.

## Conflict of interest statement

The authors declare that the research was conducted in the absence of any commercial or financial relationships that could be construed as a potential conflict of interest.

