# Supplementary figures and images for: Anti-emetic Action of the Brain-Penetrating New Ghrelin Agonist, HM01, Alone and in Combination With the 5-HT3 Antagonist, Palonosetron and With the NK1 Antagonist, Netupitant, Against Cisplatin- and Motion-Induced Emesis in Suncus murinus (House Musk Shrew)
Source: Front Pharmacol. 2018 Aug 6;9:869. doi: 10.3389/fphar.2018.00869 (PMC6087754; doi:10.3389/fphar.2018.00869)

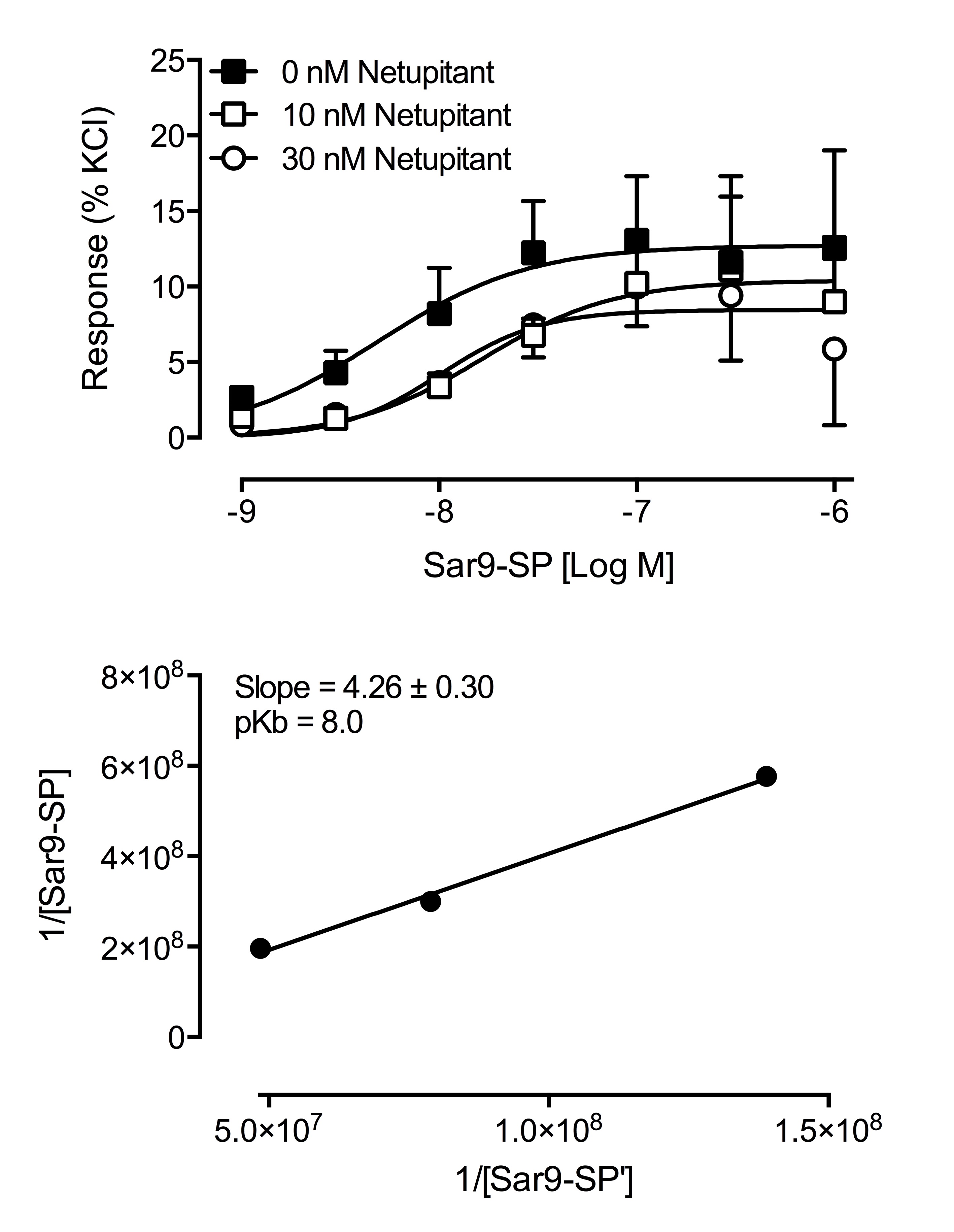

Supplement: FIGURE S1 — Effect of netupitant on [Sar9Met(O2)11]-substance P -induced contractions of Suncus murinus isolated ileum (top); double reciprocal plot for [Sar9Met(O2)11]-substance P in the presence of 30 nM netupitant (bottom). Data represents the mean ± SEM of 6 determinations. [file Image_1.JPEG]

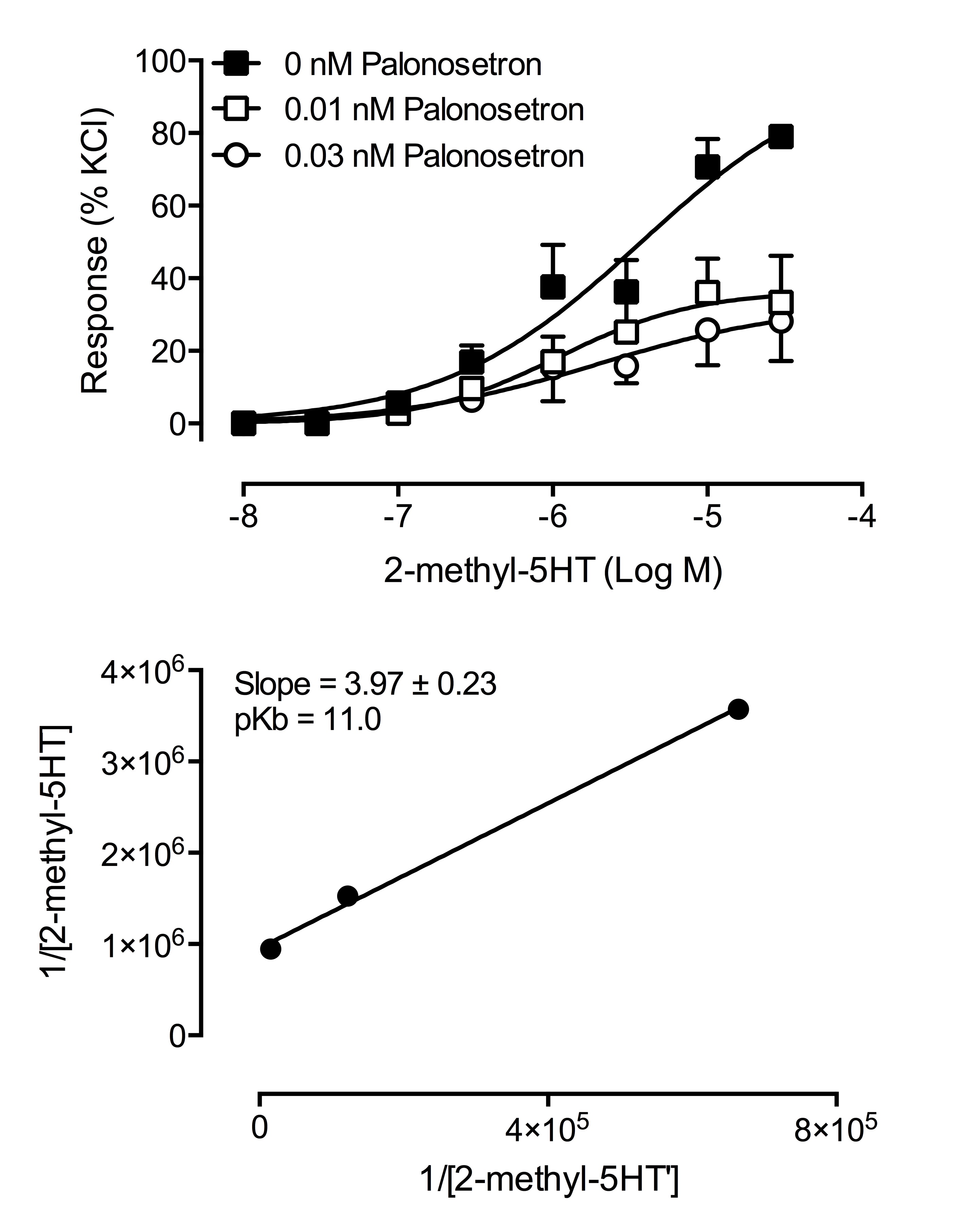

Supplement: FIGURE S2 — Effect of palonosetron on 2-methyl-5-HT-induced contractions of Suncus murinus isolated ileum (top); double reciprocal plot for 2-methyl-5-HT in the presence of 0.03 nM palonosetron (bottom). Data represents the mean ± SEM of 6 determinations. [file Image_2.JPEG]
